# A Cost-Efficient 5G Non-Public Network Architectural Approach: Key Concepts and Enablers, Building Blocks and Potential Use Cases

**DOI:** 10.3390/s21165578

**Published:** 2021-08-19

**Authors:** Panagiotis Trakadas, Lambros Sarakis, Anastasios Giannopoulos, Sotirios Spantideas, Nikolaos Capsalis, Panagiotis Gkonis, Panagiotis Karkazis, Giovanni Rigazzi, Angelos Antonopoulos, Marta Amor Cambeiro, Sergio Gonzalez-Diaz, Luís Conceição

**Affiliations:** 1 General Department, National and Kapodistrian University of Athens, 34400 Psachna, Greece; ptrakadas@uoa.gr (P.T.); lsarakis@uoa.gr (L.S.); 2 School of Electrical and Computer Engineering, National Technical University of Athens, 9, Iroon Polytechniou Str., 15780 Athens, Greece; angianno@mail.ntua.gr (A.G.); sspantideas@central.ntua.gr (S.S.); ncapsalis@gmail.com (N.C.); 3 Department of Informatics and Computer Engineering, School of Engineering, University of West Attica, 12243 Athens, Greece; p.karkazis@uniwa.gr; 4 Research and Innovation Department, i2CAT Foundation, Gran Capità 2-4 Edifici Nexus I 2ª Planta, 08034 Barcelona, Spain; giovanni.rigazzi@i2cat.net; 5 Research and Innovation Department, NearbyComputing S.L., Travessera de Gràcia 18, 3r, 3a, 08021 Barcelona, Spain; aantonopoulos@nearbycomputing.com; 6 Research and Innovation Department, Nemergent Solutions SL, Ribera de Axpe, 6, 48950 Erandio, Spain; marta.amor@nemergent-solutions.com; 7 Research and Innovation Department, Atos, Calle Albarracin 25, 28037 Madrid, Spain; sergio.gonzalez.diaz@atos.net; 8 Research and Innovation Department, Ubiwhere, Tv. do Sr. das Barrocas 38, 3800-075 Aveiro, Portugal; lconceicao@ubiwhere.com

**Keywords:** 5G, private networks, O-RAN, network monitoring and telemetry, AI/ML based network optimization, time sensitive networking

## Abstract

The provision of high data rate services to mobile users combined with improved quality of experience (i.e., zero latency multimedia content) drives technological evolution towards the design and implementation of fifth generation (5G) broadband wireless networks. To this end, a dynamic network design approach is adopted whereby network topology is configured according to service demands. In parallel, many private companies are interested in developing their own 5G networks, also referred to as non-public networks (NPNs), since this deployment is expected to leverage holistic production monitoring and support critical applications. In this context, this paper introduces a 5G NPN architectural approach, supporting among others various key enabling technologies, such as cell densification, disaggregated RAN with open interfaces, edge computing, and AI/ML-based network optimization. In the same framework, potential applications of our proposed approach in real world scenarios (e.g., support of mission critical services and computer vision analytics for emergencies) are described. Finally, scalability issues are also highlighted since a deployment framework of our architectural design in an additional real-world scenario related to Industry 4.0 (smart manufacturing) is also analyzed.

## 1. Introduction

The widespread rollout of fifth generation (5G) networks is not very far from becoming a reality [1,2,3,4,5,6]. In such networks, there are three types of services whose requirements guide the technological evolution: massive machine type communications (mMTC), supporting millions of Internet of Things (IoT) devices with intermittent activity and transmission of small data packets [7], ultra-reliable and low-latency communications (URLLC), allowing zero-latency communication with high reliability, such as in critical and emergency applications as well as communications among connected vehicles [8], and enhanced mobile broadband (eMBB), accommodating traffic with high data rates, as well as cell-edge users’ connectivity [9]. As the 5G era introduces various advantages towards providing ubiquitous coverage with high data rate availability, densification and high capillarity of access points are required to enhance 5G system capacity [10,11,12,13]. In this context, a density of (even) thousands of nodes (drones or cars equipped with remote radio-head (RRH) or other 5G network modules) per square kilometer can be achieved, while in a traditional mobile network, there are usually not more than twenty base stations in the same area. A technical overview of the most significant challenges related to network densification can be found in [14]. To this end, the deployment of a large number of smallcells usually requires locations with costly backhaul and power facilities, which might hinder the mass deployment of smallcells. In addition, it should be highlighted that the deployment of “parallel” access networks would be very economically inefficient, especially in dense areas. Within future 5G infrastructures, network sharing should evolve beyond the traditional infrastructure sharing models used in previous generations, towards cloudification and virtualization. This is related to the need for multi-operator and neutral host shared infrastructure together with the higher network capillarity due to increasing number of users and particularly the exponential growth of connected things for IoT.

Device manufacturers and vendors are already deploying 5G network equipment while mobile network operators (MNOs) are on the verge of commercializing 5G networks. MNOs have built their business almost entirely on public outdoor networks, offering access, connectivity, and managing large amounts of mobile data everywhere, supporting new services and content, while indoor (home and enterprise) services are covered with faster fixed lines (i.e., fiber optic) and Wi-Fi spots [15]. For this reason, many companies are interested in building their own 5G private networks (PNs), customized to optimally serve their specific use cases. To this end, a PN is a local area network that provides dedicated bandwidth using 5G technology. It can be deployed for a specific industrial application or for multiple industrial applications with diverse requirements. PNs are also referred to as non-public networks (NPNs) according to 3GPP [16].

Although the concept of dedicated PNs for large enterprises or industries is not a new one, the advent of 5G networks provides plenty of new business opportunities for multiple actors [17]. Indeed, 5G PNs are expected to deliver high-speed, low-latency, and other 5G benefits, supporting next-generation applications. In this context, in [18], a technical overview of private 5G networks is provided, while spectrum opportunities and design challenges are discussed as well. In [19], a private mobile edge cloud was proposed to overcome the limitations of cloud-centered and edge computing systems. Based on the proposed framework, a number of bandwidth-demanding applications, such as pervasive video, virtual reality, and mobile video surveillance can be supported. In [20], a number of deployment options for NPNs in the industry 4.0, based on 3GPP 5G specifications, is described. In this context, a comparative analysis of these options is provided as well, assessing their feasibility according to different criteria, including technical, regulatory, and business aspects. In [21], the impact of 5G networks in a manufacturing environment is analyzed. To this end, key feasibility features related to scalability, security, and data analytics are described. In [22], the authors provide an integrated methodology and approach for benchmarking and profiling 5G vertical industries’ applications. To this end, this approach will facilitate the development of artificial intelligence (AI) models in industrial environments. In [23], the authors present a time sensitive networking (TSN) implementation for a 5G network operating at 28 GHz, in the context of an NPN. Moreover, NPN and TSN assisted 5G catering for smart systems, such as the smart home or vehicle, is emulated.

In parallel, there is a growing interest towards the adoption and implementation of open solutions and interfaces towards the design of NPNs in order to leverage the reusability and scalability of private 5G architectures. This is expected to leverage the concept of cell densification, as previously described. A typical example is the Open RAN (O-RAN) alliance, primarily aiming at the adoption of standards that are fully supporting and complimentary to the ones promoted by 3GPP and other industry standard organizations [24]. Building on the principles of openness and disaggregation of the RAN component, O-RAN deployments will be crucial to overcome the current issues with vendor lock-in and are expected to be the key pillar for the realization of cost-efficient and scalable NPNs. At the same time, additional key enabling technologies, including (i) the support of data analytics and AI/ML-based network optimization, (ii) RAN sharing and neutral hosting, (iii) resource and service orchestration over heterogeneous infrastructures, as well as (iv) the support of TSN, particularly for industrial applications, will primarily drive the technological evolutions in NPN design and deployment.

Driven by the above-mentioned considerations, the goal of this paper is to propose an architectural approach for the deployment and support of 5G PNs. This architecture is fully exploitable and open by adopting RAN functions on open interfaces and standard hardware platforms. At the same time, the support of innovative verticals is achieved through cloud native orchestrators, managing the various IoT elements and customized devices, as well as the migration of virtual network functions (VNFs) across different network nodes [25]. In the proposed system, intelligent management of the network, the infrastructure resources, and the services are facilitated by AI and machine learning (ML) algorithms and the provision of data analytics from all architectural layers. The overall goal is to provide cost-efficient deployments of 5G PNs able to support a variety of applications and use cases with distinct characteristics in terms of throughput, latency, number of supported user equipments (UEs) and sensitivity to timing inaccuracies, among others. In addition, to address the diverse requirements of different types of 5G services, the system includes a network slicing component, which can exploit AI/ML functionality and provide end-to-end (E2E) slices in the compute, network, and access network domains. Targeting PNs with reduced deployment and operational costs, our system also supports neutral host RAN sharing. The required resource and service orchestration in our approach is undertaken by an E2E solution operating on top of 5G infrastructures, composed of heterogeneous components like virtual network functions, multi-access edge computing (MEC) resources and hardware devices. The orchestration solution is aligned with the latest O-RAN specifications and compatible with the relevant interface for managing the O-Cloud infrastructures.

The rest of this manuscript is organized as follows: Key scientific challenges in the design and implementation of private 5G networks are provided in Section 2. The proposed architectural approach is described in Section 3 in high level analysis, while Section 4 analyzes the proposed architectural layers along with specific components. In Section 5, three indicative use cases are described. Finally, concluding remarks are provided in Section 6.

## 2. Key Concepts and Enablers for 5G Private Networks

According to the target key performance indicators of future networks, an efficient 5G network deployment should be able to support high data rate services in diverse geographical areas. Therefore, the concept of cell densification (i.e., placement of a sufficient number of cell sites in order to increase overall capacity) is expected to significantly boost the achievable rates and, at the same time, to support zero-latency applications. However, it may be expensive for all mobile operators serving a specific area to fully deploy a dense small cell network in the same geographical area, mainly due to the presence of inter-operator coordination issues. Moreover, in the case of outdoor small cell deployments, there might be quantitative restrictions posed by local regulations regarding the technical specifications and/or the number of possible network elements that can be deployed. In addressing this issue, the use of neutral hosts could be especially attractive in locations with physical space constraints for the deployment of multiple networks [26].

Additionally, in the general context of the network automation and zero-touch optimization, another important aspect of 5G networks is their ability to be dynamically reconfigurable according to traffic demands or in the case of severe network outage. In this case, advanced AI/ML algorithms should be in place to run on top of 5G networks in order to (i) provide a holistic network reconfiguration and (ii) assist the radio resource management when necessary [27,28,29,30]. Therefore, network monitoring and telemetry is another key aspect of future broadband wireless networks, as they enable real-time network observability and data collection. In the following subsections, an outline of the main key enablers towards the design and implementation of the proposed architectural approach for NPNs is provided.

### 2.1. Data Analytics and AI/ML-Based Network Optimization

Data collection and analysis are of primary importance in 5G networks (both public and private) due to the increasing need for efficient monitoring and management of the network and underlying infrastructures. At the same time, technologies like data analytics and AI/ML are investigated as a means to facilitate network automation and result in enhanced utilization of the network resources [31]. Network automation is particularly important for the cost-efficient deployment of NPNs since it has the potential to significantly reduce management costs. Towards this direction, AI/ML techniques are of the utmost importance for the deployment of an intelligent, cognitive, and flexible 5G system, primarily due to the memorization and accurate decision-making capabilities [32]. As a matter of fact, ML algorithms have already been proven to represent a powerful tool with many potential applications to enhance and facilitate wireless communication networks in several areas, such as radio channel modelling, channel estimation, signal detection, network management and performance improvement, access control, and resource allocation.

In our proposed architectural approach, as will be described in the following section, the system incorporates modules that are in charge of gathering information from all architectural layers and provide this information either to the registered services or to AI/ML-based modules for network and service optimization. In this context, cross-layer data gathering is supported. In addition, network and management related data are processed by AI/ML algorithms in order to optimize specific parameters and key performance indicators (KPIs). Typical examples include network configuration (RAN planning and deployment, scheduling), cross-slice optimization for improved quality of service (QoS), energy consumption by each UE, as well as mobility management.

### 2.2. Network Slicing

The term network slice refers to a composition of adequately configured network functions, network applications, and the underlying cloud infrastructure (physical, virtual, or even emulated resources, RAN resources etc.), that are bundled together to meet the requirements of a specific use case (or service level agreement (SLA)), e.g., bandwidth, latency, processing, and resiliency, coupled with a business purpose [33,34]. In 5G and beyond networks, network slicing emerges as a unique approach for segmenting the physical infrastructure into multiple isolated logical networks (i.e., slices), each one specialized to satisfy a particular service (and its respective SLA). Moreover, as analyzed in the previous subsection, different MNOs may partially or holistically share the same 5G infrastructure via the instantiation of different dedicated slices. Since the network resources for a specific slice depend highly on the time-varying user demands, a slice management entity is required to dynamically deal with the slice resource allocation.

The slicing solution specified in our proposed approach will be in position to provide E2E slices in the computation, network, and access network domains and perform slice resource partitioning at the infrastructure, network slice, and vertical service levels. Notably, the solution will include an AI component responsible for processing data analytics information provided by the system in order to predict possible scarcities in slice resources that can have an impact on the performance of the running services.

### 2.3. Neutral Hosting and RAN Sharing

One of the disruptive concepts that has evolved in the telecom industry during recent years is neutral hosting. Unlike the traditional individual deployment and operation by each MNO, a third neutral party builds and operates part of the network, offering either private or public connectivity. Hosted clients, such as MNOs, civil protection entities, or other private operators, will be able to lease the neutral host to supply their services at the network edge. In this context, one of the potential strategies for reducing deployment and operation costs is network sharing through neutral hosting [35]. The idea is to have a single network infrastructure that is owned by a third party and leased to interested operators. The neutral hosting model is especially suited to infrastructure owners of large campuses that need both indoor and outdoor coverage. Utilizing a new mid-band spectrum for 5G deployments results in hyper-dense radio networks that are very costly to deploy and operate. Therefore, the ability to share this infrastructure among multiple public and non-public network operators not only reduces relevant costs, but also results in flexible network topologies.

### 2.4. Resource and Service Orchestration

Along with the evolution in the 5G capabilities, there is also an industry-wide change towards software defined and cloud technologies, using commercial off-the-shelf (COTS) compute and networking infrastructure to (i) manage costs and expand the supplier eco-system, and (ii) enhance openness, competition, and spur innovation in the RAN and core network. In this context, there is a need for efficient management of these virtualized infrastructures, as well as versatile service orchestration.

In our proposed approach, there is an E2E orchestration solution suitable for 5G infrastructures composed of heterogeneous components, spanning across multiple domains with various underlying virtualized infrastructure management technologies. The heterogeneous components include virtual network functions, MEC resources, and hardware devices. The orchestration solution includes support for assigning a process to a specific central processing unit (CPU) core in order to provide guaranteed QoS, and for network service migration within the available core or edge infrastructure in cases of service disruptions. In addition, it is aligned with the O-RAN specifications and compatible with the relevant interfaces for managing the O-Cloud infrastructures.

### 2.5. 5G Transport and TSN Support

Our proposed architectural approach will be based on lower-layer split (LLS). This places strict requirements on the interface between the remote unit (RU) and the distributed unit (DU, fronthaul). To address this requirement, the 5G RAN transport network (TN) apart from physical media and control/management protocols includes elements that provide the required time synchronization. In addition, the TN will be used for the realization of the TSN over 5G proof-of-concept. There are significant benefits that can be achieved for industrial use cases with the introduction of TSN and 5G wireless communication, e.g., due to increased flexibility in the deployment of industrial equipment [36]. This requires 5G to provide robust support for Ethernet-TSN communication services and interworking with wired TSN networks.

### 2.6. O-RAN

The system will support disaggregated RAN with open interfaces following the O-RAN initiative to allow for flexible and cost-efficient 5G deployments in private and enterprise networks. This solution will not only facilitate the deployment of a 5G RAN using components from multiple vendors, but it will also simplify network management, leading to reduced operational costs. This will become possible by embedding intelligence using emerging deep learning techniques at both the component and network levels of the RAN architecture. In combination with the standardized southbound interfaces, AI-optimized closed-loop automation is achievable and is expected to enable a new era for network operations.

The use of standardized interfaces for O-RAN element and O-Cloud management (i.e., the cloud infrastructure addressed in O-RAN) is important to avoid vendor lock-in. In our proposed approach, the implementation of the relevant interface (O1) will allow the appropriate RAN intelligent controller to collect data from various components of the O-RAN using a standardized approach. In addition, related data can be also gathered directly from the virtualized infrastructure via the interface through which the O-Cloud management is performed (i.e., O-RAN O2).

## 3. High Level Architectural Approach

**Figure 1:**
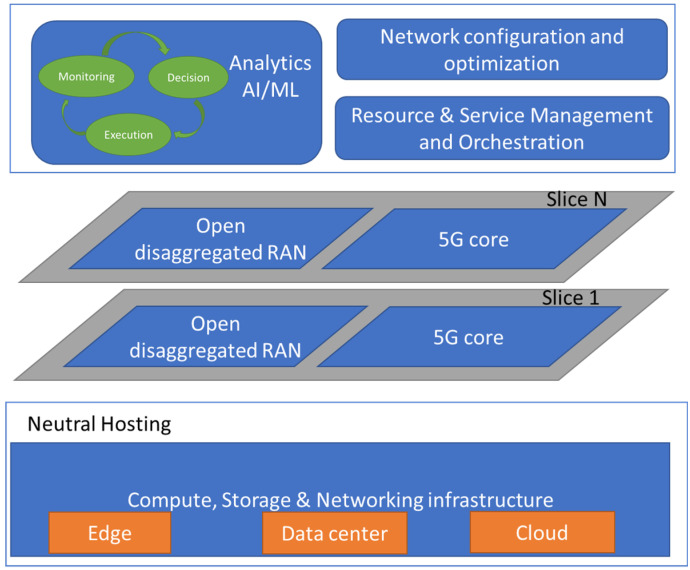
Conceptual approach of our proposed approach.

Based on the aforementioned key enablers for NPN networks, a conceptual approach of the system is depicted in Figure 1. Firstly, an identification of the involved architectural layers can be observed. At the lowest level, the infrastructure layer includes the components directly related to the actual network deployment, such as edge components, cloud infrastructure and transport network. Since individual operators need to have access to this common infrastructure in order to achieve cost-efficient network deployments, neutral hosting procedures are placed on top of these components. In the same context, as our goal is to provide various services to the end users simultaneously, different slices should be supported by the considered 5G Network. To this end, each slice has access to resources of both the 5G Core and O-RAN components of our architectural approach. The core and RAN network functions constitute the network function layer of the proposed system.

Finally, the upper architectural layer includes the proper procedures for resource and service management and orchestration, as well as network automation. In addition, as mentioned in the previous section, AI/ML algorithms target at a holistic network optimization. These algorithms gather inputs from network telemetry modules and apply dynamic network reconfigurations, depending on the network state. This functionality belongs to the management, orchestration, and automation (MOA) layer of the proposed system.

## 4. Proposed Architectural Approach

### 4.1. Architectural Components

**Figure 2:**
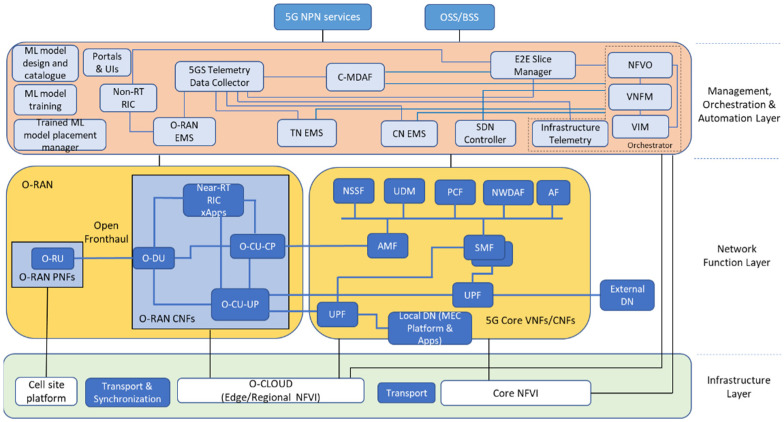
Conceptual approach of the proposed architectural concept.

The proposed architectural approach for an O-RAN-based, 5G Standalone (SA) network for NPN deployments is shown in Figure 2. The infrastructure layer consists of the core and edge/regional network functions virtualization infrastructures (NFVIs), the cell site platform, transport network segments, and synchronization elements. NFVI is a key component of the network functions virtualization (NFV) architecture that describes the hardware and software components on which virtual networks are built. The cell site platform includes proprietary infrastructures, where the software is coupled with the hardware and supports the deployment of physical network functions (PNFs).

The network function layer consists of all NFs, related either to the O-RAN or the 5G core (5GC). The O-RAN architecture is based on the decomposition of elements into NFs. To additionally support vertical disaggregation, the 5G SA core network (CN) combines the necessary NFs for the user plane (UP) and the control plane (CP).

At the MOA layer, various modules have been included providing intelligence and automation to the network and service management and orchestration. The modules are responsible to provide the following main functions:

- Resource and service orchestration
- Network slicing
- Element management
- System telemetry data collection
- Management data analytics
- ML model design, training, and placement
- Non real time O-RAN control.

The main functionalities at the MOA Layer are described in detail in the following subsections.

### 4.2. Orchestration

The NFV orchestrator (NFVO) is responsible for managing the network service (NS) lifecycle, along with the VNF lifecycle (supported by the VNF manager—VNFM), and orchestrating NFVI resources (supported by the virtualization infrastructure manager—VIM) to ensure an optimized allocation of the necessary resources and connectivity. Therefore, NFVO functions can be classified into two main categories: E2E resource orchestration, and NS orchestration. NFVO is also responsible for guaranteeing adequate NS performance and fault management, as well as VNF package management.

In the proposed approach, we provide a complete E2E orchestration solution in realistic 5G deployments that may include heterogeneous components, i.e., from virtual network functions to MEC resources and hardware devices, spanning across multiple domains with various underlying VIM technologies (i.e., Openstack and K8s). On top of these solutions, advanced AI-enabled algorithms are deployed as well in order to enable the network automation, minimizing the human intervention towards zero-touch network provisioning. Finally, it is worth noting that an integration of all deployed orchestration tools towards open and fully interoperable mobile networks is allowed through the alignment with the relevant O-RAN specifications [37]. In particular, O-RAN defines the O2 interface as an open logical interface that provides secure communication between the orchestrator and the cloud. The O2 interface enables the management of cloud infrastructures and the deployment life cycle management of O-RAN cloudified network functions that run on the cloud (at all different levels, from the edge to the central cloud).

**Figure 3:**
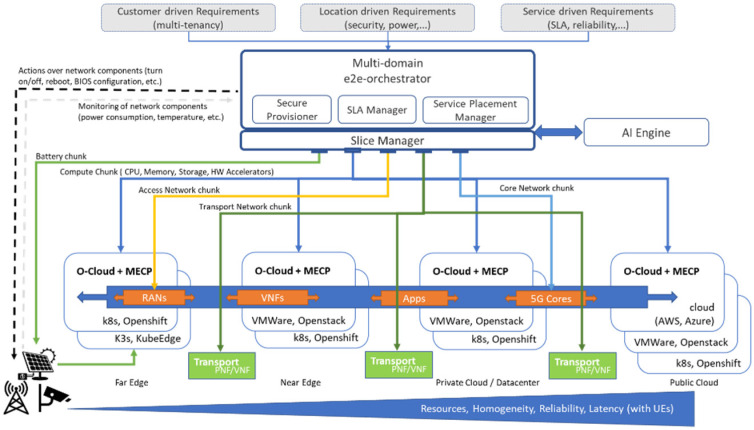
Orchestrator components.

The envisaged orchestrator’s architecture is illustrated in Figure 3. It is responsible for joint network and compute resource orchestration, actuation over network infrastructure/components and CPU pinning for isolation and guaranteed QoS. Furthermore, our developed orchestrator allows for service migration and pinning to nodes with specific hardware (HW) accelerators or features (e.g., reduced latency).

The orchestrator is composed of three main modules: (i) the secure provisioner, which provisions the node (e.g., installing any HW acceleration drivers, the operating system, etc.), (ii) the SLA manager, which is responsible for guaranteeing the SLAs of the different tenants, and (iii) the service placement manager, which decides the optimal locations for the services to be executed. In addition, the orchestrator receives different kinds of requirements and information, i.e., from customer-driven requirements (to identify the multiple tenants) to location-aware information (e.g., power constraints) and service-level KPIs. Having this information, the orchestrator is able to communicate with external entities (e.g., slice manager or AI engine) in order to make the optimal decisions during the service lifecycle.

As also depicted in Figure 3, the orchestrator can operate across multiple technical domains that, even if they are under the same administrative domain, they present significant challenges and differentiations. More specifically, the orchestrator is able to manage both network chunks (that belong either to the access, transport, or core network) and compute chunks (CPU, memory, etc.). In addition, in the compute domain, the orchestrator can handle the resources at different levels: from the public cloud, where the resources are more homogeneous and in abundance (but the delay is increased), to the far edge, where there are limited resources with high heterogeneity (but the delay is reduced).

### 4.3. Slice Manager

**Figure 4:**
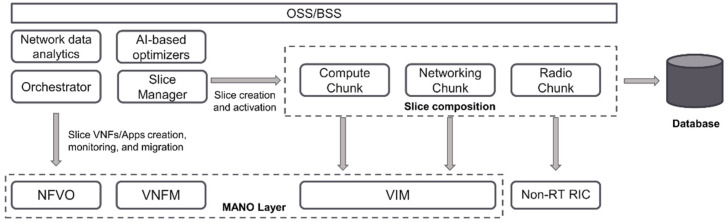
High-level view of the network slicing system.

The slice manager is responsible for the provisioning of E2E slices in the compute, network, and access network domains. In this respect, it allows several tenants to manage the required resources and to deploy services for different verticals within the slices. To accomplish its role, it monitors the performance of the slice instances and triggers slice reconfiguration actions, possibly taking into account recommendations and alerts provided by the component responsible for the analysis of the system management data. The slice manager is located at the MOA layer, as shown in Figure 4, which depicts the high-level view of this component. Within this layer, the main goal of the slice manager is the creation and management of network slice instances. Such instances are composed of three chunks of resources, namely compute, networking, and radio, as shown in Figure 4.

Moreover, the slice manager is responsible for the resource partitioning of the slices at three levels, as defined by the Next Generation Mobile Networks (NGMN) Alliance [38]: infrastructure, network slice, and vertical service. To this end, the slice manager interacts with a selected set of components at the MOA layer, as described below.

At the infrastructure level, based on the received slices requirements, the radio and computing virtualization infrastructure is created and activated through the interaction with the VIM and the non-realtime RAN intelligent controller (non-RT RIC). This operation reserves the resource chunks and associates them under a specific E2E slice ID. At network slice level, the network services required for the communication establishment of the infrastructure are instantiated. In this phase, the CN is activated, and the small cells and Wi-Fi access points start radiating. Moreover, the slice is fully activated by enabling the E2E connectivity along all respective elements. At this moment, the UEs have full inter-connectivity. Finally, the vertical service level is optional and allows third-party services to be deployed in the slice through the NFV orchestrator and the VNFM manager. In this specific case, the slice manager offloads the VNFs deployment, monitoring, scaling, and migration operations on the slices to the orchestrator.

To support cognitive functionalities, the slice manager also includes a native AI component located at the MOA layer. The main goal of this module is to analyze the information from the network data analytics function (NWDAF) included in the system. NWDAF incorporates the proper functionalities to record and offer periodic information on the status of the infrastructure, the RAN and the CN on a per-slice basis and provide concrete information on the status of the slice resources. The AI extension exploits the NWDAF-gathered data to predict possible scarcities in the resources of the slices in a specific time frame that could prevent the deployment of new services in the slice, or even to put at risk the performance of the running ones. As a result, this component provides valuable information to the slice manager in advance, raising an alarm in the system before reaching a problematic point to prevent any unwanted issue in the slice. The integration of this simple AI extension within the 5G network management platform will pave the way for further enhancements, such as the prediction of events to preempt mitigation measures, dynamic spectrum sharing, etc.

### 4.4. Network Telemetry

**Figure 5:**
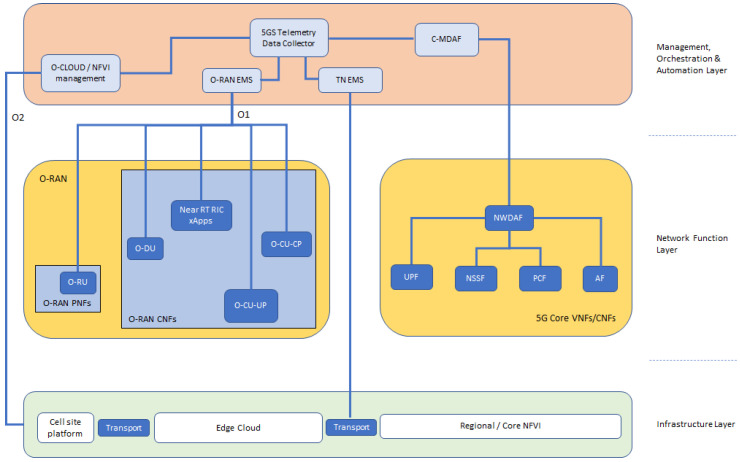
Proposed architecture for NT functionalities.

Network telemetry (NT) is real-time data collection in which devices push data to a centralized location. Telemetry metrics are generated from enterprise resources, such as switches, routers, wireless infrastructure and IoT systems, and used by business and technology applications to monitor trends and help IT respond to threats or react to changing network conditions [39]. As depicted in Figure 5, the NT framework provides analytics information for different analytic events to NF consumers, by subscribing to (1) one-time notification, (2) periodic notification, or (3) notification when an event is detected, either in the form of statistical information concerning past events, or as predictive information. In our proposed approach, NT will be able to gather related data from all defined architectural layers. In this context, two significant NT components are determined, namely the NWDAF and the centralized management data analytics function (C-MDAF), where the first one is a well-specified component of the 5G CN according to 3GPP standards and ETSI technical documents.

The NWDAF is a new feature of 5G networks, which enables the network operators to either implement their own ML-based data analytics methodologies or integrate third-party solutions to their networks. NWDAF, which is defined in 3GPP TS 29.520 [40], incorporates standard interfaces from the service-based architecture to collect data by subscription or request data from other network functions and similar procedures. The NWDAF in the 5GC plays a key role as a functional entity that collects KPIs and other information about different network domains and uses them to provide analytics-based statistics and predictive insights to 5GC network functions, for example to the policy control function (PCF). Advanced ML algorithms can utilize the information collected by the NWDAF for tasks such as mobility prediction and optimization, anomaly detection, predictive QoS and data correlation [41].

In order to support a wide variety of services and requirements and increase the flexibility, a centralized telemetry node should take advantage of management data analytics services (MDAS) towards improving the network-wide performance and efficiency [42]. These services are provided by the management data analytics function (MDAF). In principle, an MDAS offers data analytics of diverse network parameters including resource management information for a specific NF (e.g., NF’s load, resource usage status of an NF, etc.). Since the NWDAF can offer telemetry capabilities only on the CN side, the inclusion of an additional MDAF entity in the proposed architecture could extend the NT functionality towards the RAN and TN sides, as well as inter-connect the RAN, TN, and CN-oriented telemetry.

Towards this direction, C-MDAF is provisioned to host all the centralized telemetry capabilities and to be located at the MOA Layer (Figure 5). A particular NF may subscribe to the C-MDAF as a consumer in order to collect or provide data for forecasting or resource usage information purposes. Moreover, the data analysis can also identify corrective actions in the network, e.g., scaling of resources, load balancing of traffic, etc.

### 4.5. Support of AI/ML in O-RAN

O-RAN working groups have specified RAN components to host ML functionality across network domains, attending to both offline and online training and inference. Offline training refers to the time-consuming processes required for training a model. This is especially the case for deep learning-based models (or, in general, supervised and unsupervised learning), which use historically gathered data to construct predictive models. Online training refers to real-time agents that learn by interacting with the environment through trial-and-error, which is usually the case for Reinforcement Learning algorithms. The offline training support is essential in O-RANs because time-sensitive decisions have to exploit already pre-trained and validated models. In this respect, this approach mainly relies on the following design principles: (i) an offline learning module is by default essential, (ii) offline training refers to a pre-trained model that may be inferred during the online operation of the network, (iii) online training refers to the concept of real-time learners (e.g., reinforcement learning, actor-critic algorithms), as the model is trained through interaction with the network and (iv) completely untrained models cannot be directly deployed in the network without prior training and testing.

**Figure 6:**
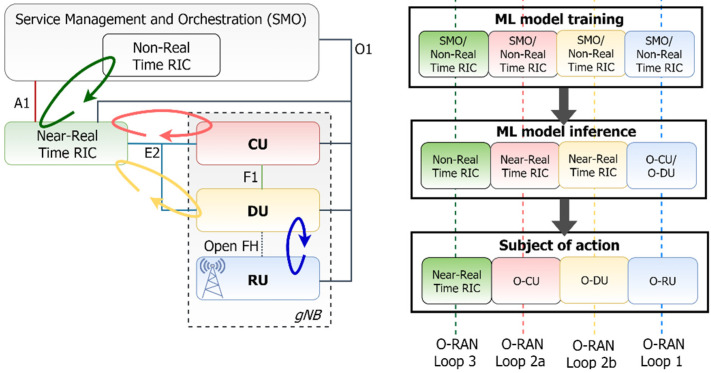
Matching the O-RAN architecture with the ML workflow ([43]).

In O-RAN based architectures, two critical building blocks will be responsible for the execution of the ML workflow: (i) the Near-RT RIC is a logical function that enables near-real-time control and optimization of RAN elements and resources via fine-grained data collection and actions over E2 interface, as depicted in Figure 6, and (ii) the Non-RT RIC, that enables non-real-time control and optimization of RAN elements and resources, AI/ML workflow including model training, inference and updates, and policy-based guidance of applications/features in Near-RT RIC (via A1 interface). It should be noted that in the deployment of supervised and unsupervised ML algorithms, the ML training host is essentially located in the Non-RT RIC, while the ML model host/actor can be located either in the Non-RT RIC or in the Near-RT RIC. On the contrary, in the framework of reinforcement learning, both the ML training host and the ML inference host/actor shall be co-located as part of Non-RT RIC or Near-RT RIC.

There are three types of control loops defined in O-RAN depending on the time sensitivity of the required decision-making process. Graphically, Loop 1 refers to the case whereby the model inference is hosted in the O-DU and the exact configuration of this loop is under consideration. Loops 2 and 3 are clearly defined as the loops that host the ML training at the Non-RT RIC, while the ML inference is typically running in the Near-RT RIC and the Non-RT RIC, respectively (Figure 6). In this context, the proposed approach implements a complete training–inference–retraining cycle, tested and checked within the whole parts of the O-RAN architecture. To this end, a holistic testing of ML models is supported, starting from the model deployment and training, model inference and action taking, and ending at the model evaluation for possible retraining and update.

## 5. Potential Use Cases

In this section, three potential use cases are described which have been selected because they are highly representative in terms of system performance, scalability, and impact in the future 5G mass market. Moreover, the peculiarities of these applications will permit to characterize the efficiency of the edge agility paradigm within different mobility patterns, slice types, deployment requirements, and triggering events.

### 5.1. Emergency Communication Critical System

This use case aims at demonstrating the robustness or “criticality” of an emergency communication system under different conditions and potential mission stages. Currently, first-responders face very distinct and unpredicted network conditions that may drastically vary over time. The 3GPP has done a great effort on standardizing the mission critical push-to-talk (MCPTT) service. This effort aims for 5G broadband networks to provide push-to-talk voice communications that get closer to the performance of private mobile radio or land mobile radio (PMR/LMR) voice, while at the same time they improve deployment time, interoperability, flexibility, and innovation. The final objective is to offer a cost-effective, open, interoperable alternative to legacy mission critical (MC) networks, while paving the way to the upcoming data and video services with broadband requirements. This use case can demonstrate the 5G NPN concept, allowing the owner to control its 5G network to serve a limited geographic area with optimized services using dedicated equipment.

The key idea behind this use case is to have a responsive service that is able to cope with drastic network conditions, so that the first-responders are able to keep communicating regardless of the outage, communication demand increase, detection of poor communication quality and so forth. The coexistence of ordinary communications by other potential network consumers in the area should be taken into consideration, requiring to differentiate between the needs that each group has in terms of QoS and priority among other features.

**Figure 7:**
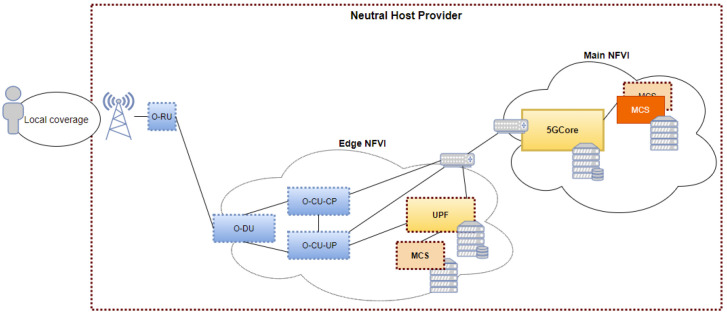
First deployment scenario of the emergency communication critical system.

**Figure 8:**
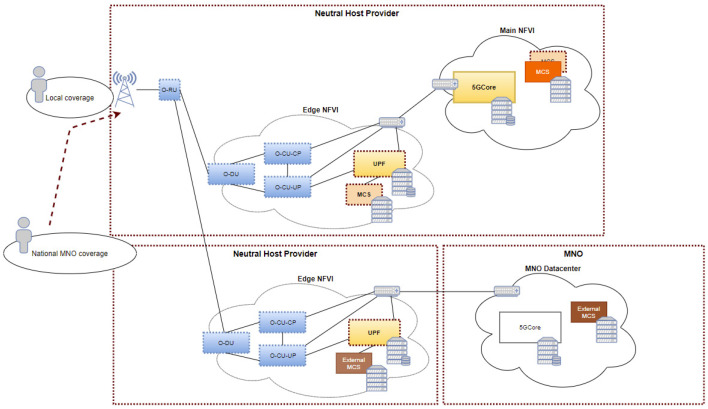
Second deployment scenario of the emergency communication critical system.

Based on the above, the first deployment scenario (Figure 7) of this use case describes a private 5G network providing services to various tenants or authorities in a concrete coverage area. The second one (Figure 8) intends to respond to an emergency situation in which national external authorities would access the concrete coverage area either by their own commercial network (in the case private and public coverages would overlap) or by attaching to the network already existent in the area. In this case the coexistence of a public 5G network to cover the needs of regular consumers and national authorities in the same coverage area where the private 5G network is deployed, will be considered in order to differentiate between the needs that each user, tenant or authority has in terms of QoS and priority among other features. In order to cope with the diverse requirements of first-responders, the use cases aim to deliver the emergency communication critical system as a private “pop up network”, providing security and privacy, control and flexibility, leveraged by network slicing, vast bandwidth, light costs, and low latency.

Depending on the executed use case, the required network service functions and application components will be instantiated in the main and edge NFVIs. When network and application services are being onboarded, the slicing will take place so the required resources allocation across the network can occur. For this use case, a network slice per tenant or authority using mission critical services will be necessary. In that way, resource availability and mission critical services’ 5G QoS mapping will be guaranteed.

Although the scope of this use case is not to put into competition diverse mission critical instances, but to monitor the performance of the application itself and to trigger application self-healing mechanisms depending on the network conditions, several slices could be created, across both main and edge NFVI, in order to observe the priority behaviour of flows within the slice. The application metrics that could be reported from the mission critical service to the monitoring module, NWDAF, include among others: users in a call, registered users in the service, number of private calls, number of group calls, maximum number of potential dialogs, etc.

### 5.2. Smart City Video Surveillance over 5G

**Figure 9:**
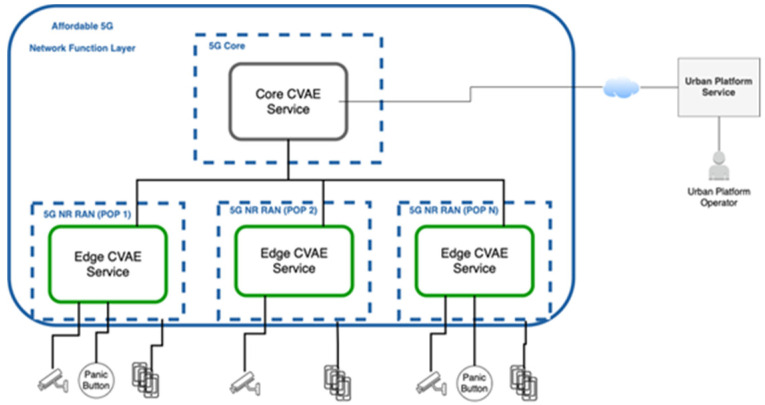
CVAE High Level Overview.

A critical requirement for the implementation of an autonomous video surveillance smart city solution is the capacity to capture, stream and process video with a high-quality level. To deploy such a solution across a wide area, a high amount of network and computational power is required to handle such a number of video streams. From its early stage, media handling has been one of the main concerns of 5G research activities associated with one of its pillar KPIs, namely eMBB. Figure 9 describes the potential video surveillance solution from a high-level perspective.

As presented in Figure 9, the computer vision analytics for emergencies (CVAE) solution is expected to deploy network services both at the core as well as the edge of a 5G ecosystem. Taking into consideration that instantiating a service at the core or at the edge pertain different requirements and limitations, as visible in the image, two kinds of CVAE network service will be developed:

Core CVAE service: This service will be instantiated at the core of the network taking advantage of all the computational power available there. This service is the central unit of CVAE solution and will be responsible for receiving and sending information from all edge CVAE service instances, deep processing of all the video streams broadcasted by edge CVAE service, correlating information sources from the different CVAE services and interacting directly with urban platform service to receive and broadcast emergency event information.

Edge CVAE service: Multiple instances of this service are expected to be instantiated at the edge of the network. Each instance is to receive the video streams of one or more closed-circuit television (CCTV) cameras and pre-process its content with the aim of detecting a potential emergency event. Additionally, these services are also prone to retrieve UE information, such as location coordinates and UE identification, as well as allow end users to manually report an emergency event. All these services are to be linked to core CVAE service to report video streams and events, as well as receive commands from it to either look for a specific object or share the video broadcast of one or more cameras connected to it.

**Figure 10:**
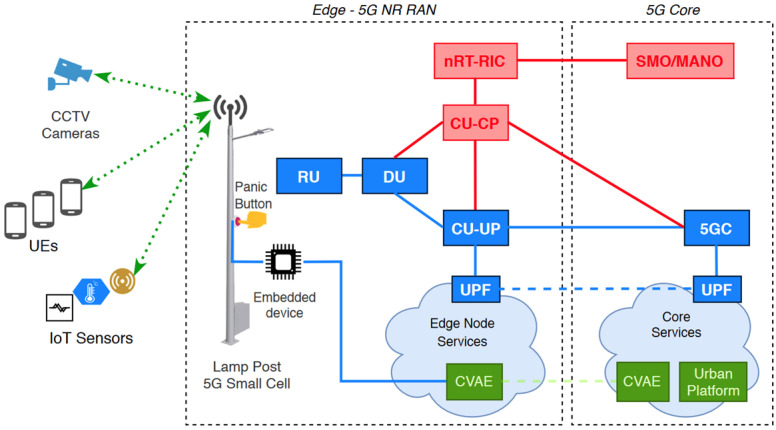
Deployment scenario.

The deployment scenario is shown in Figure 10. The end-user devices, such as CCTV cameras, UEs and IoT devices will connect to the small cell (also indicated as lamp post) via 5G new radio (NR). The lamp posts will be equipped with 5G NR RAN, computing and network resources, which will be all abstracted by a virtualized infrastructure, i.e., virtual RAN (vRAN) and virtualized infrastructure manager (VIM) in order to instantiate services at the edge node. As such, the data fed from the end-user devices flow through the DU and central unit (CU), where they can be intercepted and processed by the services running at the edge NFVI. In addition, some lamp posts will have a physical button, namely a panic button, which is hardwired to an embedded device with networking capabilities which will be linked to a particular service running at the edge. At the 5G Core a VIM will also be available to ensure the instantiation of NFV services. To be noted that (although not explicitly depicted in Figure 10) there will be multiple edge nodes connected to the 5G core, i.e., multiple lamp posts providing coverage for a certain region/location. Finally, the backhaul between the edges and the core will be assured by a fibre optic cable link.

As can be seen in the previous figure, CVAE E2E service will be comprised by a set of VNFs running both at the edge and core NFVI. CVAE E2E service expects also for a specific slice for it to be created. The network resources associated with this slice are to be readjusted to the network demands at a given point in time. In the event of one or more emergencies being detected in one more edge deployments, real time video streams are expected to be broadcasted into the core NFVI thus increasing the network bandwidth demand. Similarly, if an emergency event was a false alarm, or if it is no longer active video broadcast from edge to core is to be stopped thus releasing network bandwidth. In this context, the proposed orchestration service can handle autonomously this behaviour optimizing network resource usage.

### 5.3. Services Exploiting TSN over 5G

The term Industry 4.0 is used nowadays to refer to automation and data exchange in technologies of today’s manufacturing companies. The term was first employed by the German government as a strategy to give a modern name to the new industrial revolution, which supposes even more automation than in the third industrial revolution, adding the concept of cyber-physical systems, thanks to industrial IoT. The main aim is to reach a digital transformation with a real-time connected network able to execute autonomous decision-making processes and the corresponding monitoring of the whole system. Accordingly, 5G networks present a great challenge for these Industry 4.0 uses cases. TSN consists of a set of standards as a key enabler of deterministic and low-latency communication in the factories of the future (FoF). Further, 5G TSN is a service with high reliability and availability, capable of providing packet transport with QoS characteristics. In this context, in the following subsections, two indicative use cases are described, highlighting the need for time synchronization in NPNs deployed in manufacturing environments.

#### 5.3.1. Autonomous Mobile Robots Fleet Management

The first use case is dedicated to the benefits arising from the introduction of the TSN concept to manage autonomous mobile robots (AMRs) within a construction site. AMRs move flexibly in a hostile environment such as construction site, recognizing and avoiding obstacles at high safety levels and are easily reprogrammable. This allows AMR movement inside the whole construction in order to perform daily or specific actions. AMRs are quite useful for construction because the system can work around people and machines at high levels of safety and reduce labour needs. In addition, accident risks are reduced by avoiding risky jobs or heavy tools transport. AMRs need to trade-off operational efficiency (uptime, speed, accuracy) with safety while achieving their aim inside the construction.

#### 5.3.2. Process Automation in Factories of the Future

The second use case deals with process automation. Since the development and deployment of FoFs is inextricably connected with advances in the related fields of wireless mobile communications as well as IoT technology, the automation of various procedures, critical for the overall system operation can be supported with advanced wireless features, such as the deployment of 5G mobile networks. To this end, increased data rates and the support of zero latency applications facilitates various aspects of the production procedure. Large manufacturing companies may have dispersed production units in a territory. In the majority of involved cases, prior to the final production, various intermediate steps have to be included, namely: requirements on demand, feasibility analysis, first materials preparation, intermediate control tests, final production. Even a single failure in one of the above steps may result in product failure and related losses. Hence, product quality should be controlled throughout the production procedure, in order to minimize as much as possible potential failures. In cases of malfunction of a certain production component, feedback to the production unit should be immediate in order to pause all other related procedures.

In addition, this information should also propagate to other production units in order to avoid similar circumstances. In this context, a typical example includes companies in the printing sector since the printing process is quite complex and often requires manual interventions from the personnel. Defects along the manufacturing process have a major impact on the company’s financial losses. Therefore, rapid decisions based on immediate feedback are of the utmost importance in such a complex environment. The interconnection of sensors in the production line with the CPU can be made feasible via Ethernet cables. However, a major disadvantage of such an approach is that changes in the overall production chain would also require major modifications in the wired topology. Therefore, an alternate solution would be the wireless communication of sensors with the CPU. Although a public infrastructure can be used, private 5G networks ensure high bandwidth availability on demand (i.e., transmission of high-resolution images from the sensors) as well as latency minimization.

In this context, provision of service (network slice) may include allocation of high bandwidth channels to the sensor nodes for high resolution image transmission (uplink case) or zero latency transmission back to the nodes for decision making procedures (e.g., immediate production termination, downlink case). In addition, NWDAF can store information regarding maximum data used per session and data usage per sensor node at different times of the day. With that information, the NWDAF is able to form a data usage pattern for a node. With NWDAF input, PCF can enforce specific rules appropriately for the node according to the usage pattern. This type of proactive decision by PCF also helps in utilizing the 5G resources appropriately.

Moreover, NWDAF, apart from serving the 5G network functions, can also help potential manufacturing operators to plan their enhancement in terms of infrastructure based on resource utilization. This is useful because, based on the data provided by the NWDAF, the NFs can make real time decisions for allocating the resources, but are limited to a certain extent (based on hardware resources available).

## 6. Conclusions

In this paper, a novel architectural approach for 5G NPNs has been presented. In this context, the proposed system brings together different features and novelties targeting at efficient 5G system deployment and management. First, a key characteristic of the system is that it builds on an open, disaggregated, intelligent, virtualized, and highly extensible solution for the RAN, following the O-RAN specifications and guidelines, and leverages RAN sharing and neutral hosting.

Moreover, in the presented system, network automation and optimization are supported by data analytics modules, combining information from different NFs as well as infrastructure telemetry data. In order to provide predictions and recommendations, the data analytics modules (either operating at the management level or at the CN level) leverage appropriate AI/ML models and algorithms. To this end, and as an effort to address the lack of standardized interfaces for data collection for analytics purposes, as well as the delivery of analytics services, the 3GPP NWDAF has been used as part of the 5G core architecture.

Furthermore, the system incorporates a complete E2E orchestration solution in real 5G infrastructures, composed of heterogeneous elements, as well as the support of advanced synchronization features in the infrastructure layer, such as the TSN concept. Finally, specific real world use cases were provided, demonstrating the applicability of our proposed approach in diverse bandwidth and latency demanding applications.

## Data Availability

Not applicable.
